# Emerging Diagnostics and Therapies in Neuroendocrine Neoplasms: A Critical Review

**DOI:** 10.3390/cancers17223632

**Published:** 2025-11-12

**Authors:** Jorge H. Hernandez-Felix, Monica Isabel Meneses-Medina, Rachel Riechelmann, Jonathan Strosberg, Rocio Garcia-Carbonero, Jaydira del Rivero

**Affiliations:** 1 Developmental Therapeutics Branch, National Cancer Institute, National Institutes of Health, Bethesda, MD 20892, USA; jorge.hernandezfelix@nih.gov; 2 Department of Hematology and Oncology, Instituto Nacional de Ciencias Medicas y Nutricion Salvador Zubiran, Mexico City 14080, Mexico; mimm2404@hotmail.com; 3 Department of Clinical Oncology, AC Camargo Cancer Center, Rua Antonio Prudente 211, Sao Paulo 01509-010, SP, Brazil; rachel.riechelmann@accamargo.org.br; 4 Department of Gastrointestinal Oncology, H. Lee Moffitt Cancer Center and Research Institute, 12902 Magnolia Drive, Tampa, FL 33612, USA; jonathan.strosberg@moffitt.org; 5 Department of Medical Oncology, Hospital Universitario 12 de Octubre, Imas12, Medicine Faculty, Universidad Complutense Madrid (UCM), 28041 Madrid, Spain; rgcarbonero@gmail.com

**Keywords:** neuroendocrine tumors, radionuclide therapy, targeted therapies, PRRT, theranostics, innovations

## Abstract

**Simple Summary:**

Neuroendocrine neoplasms are uncommon cancers that arise from hormone-producing cells and can appear in many organs, making them difficult to diagnose and treat. Over the past few years, major advances have emerged in specialized imaging, blood-based molecular diagnostics, and targeted therapies such as cabozantinib and belzutifan. Novel radioactive agents, cell-based treatments, and immunotherapeutic approaches are progressing swiftly from research settings into clinical trial phases. Given the wide array of sources reporting these advancements, healthcare professionals and researchers may find it challenging to stay informed about the latest evidence. We review the most important developments up to mid-2025, discuss how each innovation enhances diagnostic and treatment capabilities, and outline ongoing studies that hold promise for further advancing patient care.

**Abstract:**

Neuroendocrine neoplasms (NENs) are biologically diverse tumors. This article is a critical review of recent evidence focusing on systemic therapies (through mid-2025). We summarize what is most practice-relevant and where gaps remain. In diagnosis, somatostatin-receptor PET/CT has largely replaced older scintigraphy, and adding FDG PET can flag more aggressive disease. Blood-based tests and selected tissue markers (e.g., MGMT, DAXX/ATRX/ALT) show promise but require cautious interpretation in routine care. In treatment, radioligand therapy (PRRT) is used earlier in appropriate receptor-positive disease; cabozantinib improves progression-free survival after prior therapy; and belzutifan offers a biomarker-guided option for malignant pheochromocytoma/paraganglioma. Immunotherapy remains limited to defined subsets, including high-grade neoplasms. We appraise strengths and limitations of key trials, note issues of access and toxicity, and highlight active areas in development (SSTR antagonists, alpha emitters, and dose-guided PRRT). Our goal is to provide a concise, evidence-based map of the field to support informed clinical judgment and future research priorities.

## 1. Introduction

Neuroendocrine tumors (NETs) are heterogeneous malignancies that originate from the neuroendocrine cells throughout the body. In the United States, the reported incidence has increased from 1.64 cases per 100,000 population in 1975 to roughly 8.52 per 100,000 in 2021, largely due to advancements in detection and an increasing recognition of disease prevalence [1,2]. The estimated 20-year prevalence of NETs exceeds 240,000 individuals, emphasizing the significance of survivorship-related considerations. Nevertheless, significant variations exist, with median overall survival of approximately 11.8 years across the general patient population; however, outcomes vary notably depending on tumor origin, for instance, a 10-year survival rate of 51.7% for well differentiated distant stage (metastatic) small-bowel primaries compared to 15.4% for rectal primaries [2].

Therapeutic options have expanded in parallel with radiotheranostics and mechanism-based agents. Notably, on 26 March 2025, the U.S. Food and Drug Administration approved cabozantinib for use in unresectable or metastatic pancreatic and extrapancreatic NETs following prior therapy [3]. Subsequently, belzutifan, a selective inhibitor of hypoxia-inducible factor-2α, received approval for the treatment of malignant pheochromocytoma and paraganglioma, introducing a novel metabolic approach to NET therapy [4]. Concurrently, peptide receptor radionuclide therapy (PRRT) is expanding beyond ^177^Lu-DOTATATE to incorporate somatostatin receptor antagonists and alpha-emitting isotopes. Furthermore, emerging research involving viral-primed immunotherapy, bispecific antibodies, and chimeric antigen receptor (CAR) T-cell therapies has the potential to redefine the systemic treatment landscape for NETs.

Despite significant progress, critical challenges remain. Access to advanced imaging techniques and PRRT varies globally; reliable predictive biomarkers for optimal treatment sequencing are still under development; and many phase III clinical trials continue to enroll patients who have undergone multiple prior therapies, which limits insights into first-line treatment strategies. In this review, we summarize developments through 2025, covering diagnostic imaging, approved and emerging systemic therapies and PRRT innovation. We outline future directions, such as precision dosimetry, cell-based strategies, and adaptive trial designs.

## 2. Diagnostic Advances: Molecular Markers, Liquid Biopsy, and Functional Imaging

**Biochemical Biomarkers**: Traditional biomarkers such as chromogranin A (CgA) are commonly used; however, they present notable limitations. While CgA levels are generally associated with tumor burden and differentiation status, up to 30–50% of patients with neuroendocrine neoplasms may exhibit normal CgA values, which can impact diagnostic and monitoring accuracy [5,6,7]. Factors such as proton pump inhibitor use and certain medical conditions can lead to false-positive results [8]. As a result, the sensitivity of CgA testing is moderate, approximately 56% according to some studies, and its overall specificity decreases to approximately 55–64% when accounting for potential confounding factors [9,10]. The current clinical guidelines recommend that treatment decisions should not be based solely on CgA levels [11]. Several multianalyte blood assays (e.g., the NETest) have been explored as adjuncts to conventional biomarkers; however, variable specificity, heterogeneous cut-offs [8] and limited prospective validation [12] currently confine their use to research settings rather than routine practice.

Circulating tumor DNA (ctDNA) is an emerging biomarker with potential clinical utility. It offers a noninvasive method to detect tumor-specific genetic alterations and methylation patterns [13]. Preliminary data indicates that the presence of ctDNA correlates with higher tumor grade and metastatic disease [14]. Specifically, ctDNA levels can differentiate metastatic from localized pancreatic NETs and are elevated in well-differentiated G3/NEC cases [15]. Serial measurements of ctDNA may serve as a useful tool for monitoring tumor burden and assessing treatment response; for example, reductions in ctDNA levels have been associated with prolonged progression-free survival in patients receiving therapies such as everolimus [14]. However, it is important to note that the sensitivity of ctDNA detection in indolent, low-burden NETs remains limited, particularly in well-differentiated G1–G2 tumors that tend to shed minimal DNA [16]. The relatively low mutation burden typical of well-differentiated NETs further restricts the utility of ctDNA in these cases.

Currently, ctDNA assays are most effectively employed in patients with advanced or high-grade disease and are primarily used within research contexts.

**Histopathology and Molecular Markers:** Tumor grading by the Ki-67 proliferation index is the cornerstone of NET diagnosis and prognosis, and it forms the basis of WHO grade stratification (G1 < 3%, G2 3–20%, G3 > 20% Ki-67-positive cells) [17]. This grading has direct therapeutic implications: the strongest antiproliferative evidence for somatostatin analogs is in Ki-67 ≤ 10%; by contrast, poorly differentiated neuroendocrine carcinomas (NECs, often Ki-67 > 50%) are biologically distinct and are generally managed with cytotoxic chemotherapy [18,19]. An accurate Ki-67 assessment is therefore critical for treatment selection. However, Ki-67 indexing is prone to inter-observer variability and sampling error [20]. Therefore, the trend is toward more objective quantification, AI models for tissue classification or double-staining techniques are needed to improve consistency [21]. Clinicians should be aware of this variability and interpret grades within clinical context when guiding treatment decisions [22,23].

Overall, somatic next-generation sequencing of NETs has offered little value in clinical practice. But recent research has reported on new potential biomarkers, such as *DAXX/ATRX* gene status and alternative lengthening of telomeres (ALT), which can enhance risk stratification in pancreatic NETs (pNETs). *DAXX/ATRX* inactivation occur in approximately 40–45% of these tumors [24] and result in loss of nuclear protein expression by immunohistochemistry (IHC), concurrently activating the telomere-lengthening ALT pathway [25]. These prognostic signal is strongest in non-metastatic pNETs; where *DAXX/ATRX* loss and/or ALT positivity is associated with increased likelihood of metastasis and reduced survival after surgery, in some cohorts independent of tumor grade [26,27,28]. Incorporating *DAXX/ATRX* status into pathology reports can improve risk stratification and identify “high-risk” pNETs that may warrant enhanced surveillance or consideration of adjuvant therapy.

A whole-genome sequencing study of 102 sporadic pancreatic PanNETs has reshaped the understanding of these tumors’ origins, indicating that they are not solely somatic in nature. Approximately 6% of cases were found to harbor pathogenic germline variants in DNA repair or tumor suppressor genes such as *MUTYH*, *CHEK2*, *BRCA2*, *MEN1*, and *VHL*. The somatic alterations observed tend to cluster into four interconnected oncogenic pathways: (1) chromatin remodeling and modification driven by mutations in *MEN1*, *DAXX*, *ATRX*, *SETD2*, or *MLL3*; (2) impaired DNA damage repair mechanisms involving base-excision or homologous recombination genes; (3) activation of the PI3K–mTOR signaling axis through loss of PTEN or DEPDC5, or via activating mutations in PIK3CA, TSC1, or TSC2; and (4) abnormalities in telomere maintenance, particularly associated with DAXX or ATRX loss [29]. These pathways collectively account for nearly 80% of the mutational landscape observed in PanNETs. This classification has limited clinical utility given its lack of prognostic value, emerging evidence indicates that germline pathogenic variants in MUTYH, often accompanied by loss of heterozygosity (LOH) of the wild-type allele; are associated with an aggressive, high-grade phenotype in metastatic pancreatic neuroendocrine tumors (PanNETs) [30]. Many of these genetic alterations remain under investigation as potential therapeutic targets. Currently, clinical trials exploring PARP inhibitors for tumors with CHEK2 or BRCA2 mutations are just beginning to emerge [31].

Upon this genomic framework, Di Domenico et al. conducted an analysis of 125 PanNETs utilizing genome-wide DNA methylation arrays. Their findings demonstrate that tumors can be categorized into three distinct groups: α-like, β-like, and intermediate. The α-like group shows enrichment for ARX expression, mutations in DAXX/ATRX, and chromosomal instability, whereas β-like tumors maintain PDX1 expression and tend to have a more indolent clinical course. The intermediate group exhibits epigenetic dedifferentiation and a higher likelihood of relapse. Importantly, methylation-based classifications provide superior prediction of disease-free survival compared to single-gene markers, underscoring the potential of integrated epigenomic analysis as a valuable prognostic tool [32].

Translational efforts are beginning to couple these molecular insights with therapy selection. One example is O^6^-methyl-guanine-DNA-methyl-transferase (MGMT): loss of MGMT expression or promoter hypermethylation, seen in roughly half of PanNETs, has been associated with improved objective response rates and prolonged progression-free survival (PFS) when treated with Capecitabine/Temozolomide (CAPTEM) in the ECOG-ACRIN E2211 trial [33]. These findings suggest that the efficacy of CAPTEM may depend on deficient DNA repair mechanisms.

Additional datasets have provided further support for this association. A real-world cohort of 80 patients treated with CAPTEM demonstrated that MGMT-negative tumors, had a median overall survival of 31 months, almost twice that of MGMT-positive tumors (median survival of 17.5 months) [34]. A prospective, single-center study evaluated MGMT promoter methylation status in 22 patients with well-differentiated NETs scheduled to receive temozolomide with or without capecitabine. Only 23% of tumors exhibited promoter methylation, this subgroup experienced significantly improved PFS (data not yet mature for median PFS) and an objective response rate of 60%, compared to 30.2 months PFS and a 24% response rate in patients with unmethylated tumors [35].

The MGMT-NET trial was a randomized study designed to test MGMT as a predictive biomarker in NETs. This multicenter, open-label, biomarker-stratified randomized phase II, assigned patients with MGMT-deficient (dMGMT) or MGMT-proficient (pMGMT) disease to alkylating-agent versus oxaliplatin-based therapy, dMGMT tumors showed higher best ORR (53% vs. 12%) and longer median PFS (14.6 vs. 11.3 months) with alkylating-agent, while MGMT status did not influence oxaliplatin efficacy [36].

**Advances in Imaging:** The development of somatostatin receptor (SSTR) positron emission tomography (PET) imaging, utilizing radiolabeled somatostatin analogs such as 68Ga-DOTATATE, represents an improvement over traditional nuclear medicine techniques. Conventional scans with ^111^In-octreotide (OctreoScan) exhibited moderate sensitivity (approximately 60–80% in detecting NETs) and frequently overlooked smaller lesions [37]. In comparison, ^68^Ga-DOTATATE PET/CT offers superior resolution and increased lesion detectability. Meta-analyses estimate sensitivity and specificity to be around 90–91% for localizing NET lesions [38]. Direct comparative studies within the same patient cohorts have demonstrated that Ga-68 PET imaging identifies a greater number of lesions than ^111^In-based scans [39,40]. This accuracy directly influences clinical decision-making, as SSTR PET improves staging, can change surgical plans, and reduces false positives. Current clinical guidelines recommend SSTR PET imaging as the preferred modality for NET evaluation when available [41].

The theranostic application of SSTR imaging is a key advantage, as patients with tumors exhibiting strong uptake are ideal candidates for PRRT [42]. Imaging outcomes, such as the Krenning score, originally developed for ^111^In-based scans, serve as predictive biomarkers to determine suitability for PRRT [43,44]. In practice SSTR-positive tumors may be managed with somatostatin analogs or PRRT, while SSTR-negative tumors often require alternative therapies such as systemic chemotherapy. Common tracers include ^68^Ga- or ^64^Cu-labeled DOTATATE/DOTATOC/DOTANOC [45]. Conversely, in higher-grade or dedifferentiated tumors, SSTR expression may diminish, 18F-fluorodeoxyglucose (FDG) PET may be considered for further assessment.

The integration of dual-tracer imaging, combining SSTR PET and FDG PET, has gained recognition for capturing the diverse biological behaviors of NETs [46,47]. Well-differentiated tumors, typically show strong SSTR uptake with minimal FDG activity, whereas more aggressive or dedifferentiated tumors, often demonstrate high FDG uptake indicative of increased glycolytic activity and reduced SSTR expression [48]. The validated “NETPET” scoring system consolidates findings from both scans to serve as a prognostic biomarker; patients with FDG-positive tumors tend to have poorer survival outcomes compared to those with SSTR-positive and FDG-negative profiles [49]. Practically, an SSTR-positive/FDG-negative profile (NETPET score P1) suggests a more indolent disease course amenable to somatostatin analogs and PRRT, whereas FDG-positive lesions (scores P4–P5) signal a more aggressive phenotype requiring early systemic chemotherapy.

## 3. Current Systemic Therapies for Neuroendocrine Tumors (NETs)

**Figure 1:**
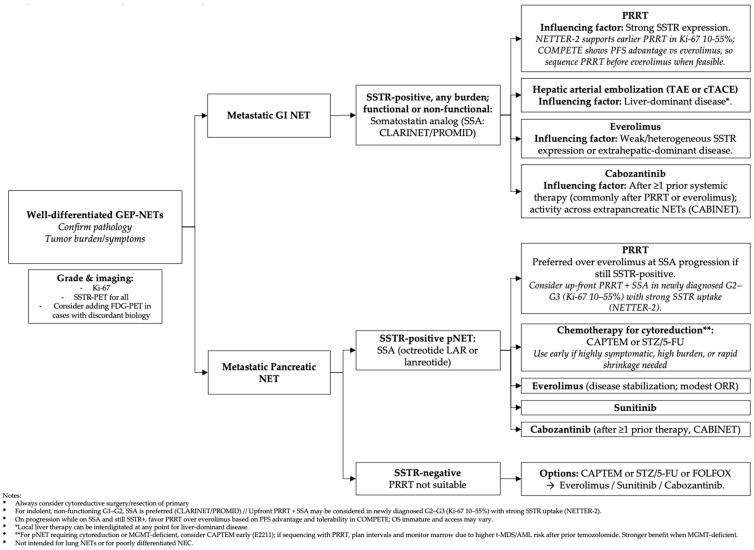
Potential algorithmic management of well-differentiated GEP-NETs as of mid 2025.

Systemic treatment selection for NETs is driven by tumor grade, SSTR expression, anatomic site, functional syndrome, prior exposure and patient-related issues (performance status and comorbidities) and preferences. Recognizing the wide biologically heterogeneity across all primary sites, this section evaluates the evidence for each therapeutic class, highlighting landmark trials, and pragmatic sequencing strategies. See Table 1 and Figure 1.

### 3.1. Hormonal Control and Symptom Management

Somatostatin analogues (SSAs) are commonly utilized as the initial systemic treatment for NET that express somatostatin receptors, owing to their established safety profile and antiproliferative properties [50]. Long-acting formulations such as octreotide (30 mg administered every four weeks) and lanreotide (120 mg administered every four weeks) effectively manage hormone-mediated syndromes and contribute to tumor stabilization in low-grade NETs. Clinical trials, including the PROMID study focusing on mid-gut NETs and the CLARINET study involving non-functioning gastroenteropancreatic NETs, demonstrated significant reductions in the risk of disease progression (hazard ratios of 0.34 and 0.47, respectively) [51,52]. These agents are generally well tolerated, with the most common adverse effects being transient gastrointestinal symptoms. While direct comparative studies between these agents are lacking, a SSA class effect assumes both drugs have equivalent efficacy, and selection typically depends on factors such as dosing and administration convenience and contractual agreements with payers.

Approximately 20% of patients may experience secondary tachyphylaxis; in such cases, dose escalation (e.g., increasing octreotide LAR to 40–60 mg or administering lanreotide every two to three weeks) or transitioning to short-acting octreotide rescue dosing can often restore therapeutic response [53,54]. When considering dose escalation beyond guideline recommendations, clinicians should carefully weigh the potential benefits against the increased risk of adverse effects, including steatorrhea, biliary sludge formation, and micronutrient malabsorption [55].

For patients experiencing refractory carcinoid syndrome associated diarrhea unresponsive to optimized somatostatin analogue therapy, the tryptophan hydroxylase inhibitor telotristat at a dose of 250 mg three times daily demonstrated a reduction in bowel movement frequency of at least 30% in 44% of participants within the TELESTAR study. Additionally, telotristat was associated with a median decrease of 40% in urinary 5-HIAA levels [56].

Telotristat is generally well tolerated; risks include depression-related symptoms (depressed mood, anhedonia, suicidal ideation) and constipation [56]. According to current clinical guidelines, telotristat should be considered as the subsequent step in therapy escalation for suboptimal control carcinoid syndrome diarrhea on somatostatin analog [55].

Paltusotine represents a promising advancement in oral SSTR2 agonist therapy [57]. Phase II clinical data indicate that once-daily administration of paltusotine achieves at least a 50% reduction in chromogranin-A or 5-HIAA levels in over 80% of patients transitioning from injectable treatments, with a tolerability profile primarily characterized by mild gastrointestinal discomfort [58]. In a randomized, parallel-group Phase II study, paltusotine demonstrated clinically significant improvements in carcinoid syndrome symptoms, including a median 63% reduction in flushing episodes, a 46% decrease in daily bowel movements, and over 70% of patients reporting overall symptom improvement on the Carcinoid Symptom Severity Scale. The treatment was well tolerated, with no grade 3 or higher drug-related adverse events reported [59]. The ongoing CAREFNDR placebo-controlled phase III trial, is anticipated to further define its therapeutic role [60]; until such data are available, paltusotine should be used exclusively within the context of clinical research settings.

Insulinoma management depends on clinical setting. For localized insulinoma, curative surgery is the treatment of choice; diazoxide is typically used as a preoperative bridge to control hypoglycemia, often with a thiazide to limit edema [55].

Short-acting octreotide can help with breakthrough episodes in SSTR2-positive tumors, but its effect is variable and a brief test is advised to avoid paradoxical worsening. Malignant insulinoma (metastatic or unresectable) care should prioritize tumor control; everolimus frequently improves hypoglycemia and is a reasonable early systemic option alongside PRRT/CAPTEM or liver-directed therapy [61]. Finally, RZ358 (an anti–insulin-receptor monoclonal antibody) has emerging evidence for severe, refractory hypoglycemia, reported in a case report [62] and in an expanded-access series [63], but remains investigational.

### 3.2. Targeted Therapies: mTOR Inhibitors and Tyrosine Kinase Inhibitors

Molecular targeted therapies have significantly advanced the management of NETs, particularly in cases of advanced disease. Everolimus, an oral inhibitor of the mechanistic target of rapamycin (mTOR), was among the first targeted agents approved following the results of the phase III RADIANT clinical trials [64]. In patients with progressive NETs, particularly in pancreatic NETs, everolimus has demonstrated a substantial improvement in PFS compared to placebo (median PFS approximately 11 months versus 4 months). Its benefits have been extended to extrapancreatic NETs, including pulmonary and gastrointestinal primaries, as evidenced by the RADIANT-4 trial, establishing everolimus as a standard second-line therapy across non-functioning NET subtypes [65]. It is important to note that objective response rates with everolimus are modest (<5%), primarily contributing to disease stabilization rather than tumor reduction. Adverse effects are dominated by stomatitis (70%; grade ≥ 3 ~7%), hyperglycemia (75%; grade 3–4 17%), and anemia (15% grade 3–4); periodic blood counts, renal function and glucose/lipids are recommended [66]. Lower doses of everolimus (5–6 mg once daily) appear similarly effective to the standard 10 mg once daily in NETs based on retrospective data [67], and this is being tested in a randomized, open-label, near-equivalence phase II trial (EVENET; NCT06472388). Currently, no definitive predictive biomarkers have been identified to select patients most likely to benefit from mTOR inhibition; while PI3K–AKT–mTOR pathway mutations (e.g., TSC1/TSC2/PTEN/PIK3CA) have shown exploratory associations, these findings are not actionable in routine care; treatment with everolimus is therefore guided by clinical factors.

Multi-targeted tyrosine kinase inhibitors (TKIs) that inhibit angiogenesis and other signaling pathways are increasingly integral to NET treatment, particularly in pNETs. Sunitinib, a vascular endothelial growth factor receptor (VEGFR) inhibitor, received approval for advanced pNETs following a phase III trial demonstrating improved PFS (approximately 11 months versus 5.5 months with placebo) and a trend toward increased overall survival [68]. Its efficacy in non-pancreatic NETs remains less well established due to limited trial data, with most evidence supporting use in pNETs. The common adverse effects include hypertension, hand-foot syndrome, and fatigue, often requiring dose adjustments.

Recently, cabozantinib has emerged as an active TKI across multiple NET subtypes. It inhibits VEGFR2, MET, AXL, and additional kinases. The phase III CABINET trial identified substantial PFS improvements in both pancreatic and extrapancreatic NET groups [69]. In this study, patients with progressive well-differentiated NETs who had received at least one prior therapy were randomized to cabozantinib 60 mg daily versus placebo; the trial was stopped early due to clear efficacy findings. In pancreatic NETs, median PFS was 13.8 months with cabozantinib versus 4.4 months with placebo, with a hazard ratio (HR) of 0.23 (*p* < 0.0001). In extrapancreatic NETs, median PFS was 8.4 versus 3.9 months, respectively (HR 0.38, *p* < 0.0001). The benefit was observed across various tumor sites, including midgut, lung, and high-grade well-differentiated NETs. Objective response rates were modest (~19% in pNETs and 5% in other NETs), but disease stabilization was the predominant outcome, with many tumors exhibiting meaningful tumor regression compared to placebo [69].

The robust design of the CABINET trial, including separate analyses for pNET and other NETs, enhances confidence in these findings. While crossover to cabozantinib upon progression likely limited the ability to detect overall survival benefits (observed HRs around 0.86 and 0.95 in different cohorts, not statistically significant), the primary endpoints were met with high statistical significance. Based on these results, the FDA approved cabozantinib in March 2025 for adult and adolescent patients (≥12 years) with advanced, well-differentiated NETs after prior therapy. This approval establishes cabozantinib as a new standard systemic therapy, although real-world implementation may be influenced by considerations such as cost and regional regulatory frameworks. clinicians should be vigilant in managing potential toxicities, as approximately 65% of patients in the trial required dose reductions due to adverse events like hypertension, diarrhea, and palmar-plantar erythrodysesthesia, and up to 20–30% discontinued therapy altogether. Careful patient selection and proactive toxicity management are essential to optimize outcomes [69].

A global randomized phase 2/3 study (STELLAR-311, NCT06943755) is evaluating the next-generation TKI zanzalintinib (XL092) versus everolimus in previously treated, well-differentiated grade 1–3 pancreatic and extra-pancreatic NETs; its results should help inform positioning relative to mTOR inhibition.

### 3.3. Cytotoxic Chemotherapy

Cytotoxic chemotherapy is generally indicated for more aggressive neuroendocrine tumor subtypes. While recent therapeutic developments have increasingly focused on targeted agents and radiolabeled therapies, cytotoxic chemotherapy remains a vital treatment option for specific neuroendocrine tumor phenotypes, particularly pNETs with high tumor burden, high-grade well-differentiated tumors (Ki-67 index between 20% and 55%), and poorly differentiated neuroendocrine carcinomas (NECs).

The combination of capecitabine and temozolomide (CAPTEM) is now considered the standard doublet therapy for progressive pNETs based on current evidence. The results from the ECOG-ACRIN E2211 trial demonstrated that CAPTEM significantly prolonged progression-free survival (PFS) versus temozolomide alone (22.7 vs. 14.4 months; HR 0.58; *p* = 0.02), objective response rates were higher but not statistically significant (40% compared to 34% with temozolomide alone) and extended median progression-free survival to approximately 22.7 months. Tumors lacking MGMT expression or with MGMT-promoter methylation showed the most pronounced responses, suggesting that immunohistochemical assessment or methylation status can be useful in patient selection. The main adverse effects observed included hematologic and gastrointestinal toxicity, grade ≥ 3 AEs occurred in 45% with CAPTEM vs. 22% with temozolomide alone, driven largely by neutropenia (8–15%), thrombocytopenia (7–10%), and anemia (5–10%). GI toxicities (nausea/vomiting 20–30%, diarrhea 10–15%, mucositis/stomatitis 5–10%) and fatigue (20–50%) are common; hand–foot syndrome occurs in 10–20%. Dose reductions are frequent, while discontinuations are relatively uncommon; rare late events such as MDS have been reported [33].

Historically, Streptozocin-based regimens (streptozocin–fluorouracil or doxorubicin) achieved response rates up to 42% in pNETs [70]. Their use has fallen because of renal toxicity, gastrointestinal side effects, and the practical demands of multi-day intravenous dosing with pre/post-hydration and laboratory monitoring; consequently, oral options are often favored when appropriate [71].

For poorly differentiated NECs and certain very highly proliferative, well-differentiated G3 NETs, platinum–etoposide doublets remain the standard first-line systemic therapy [72]. Randomized studies comparing etoposide/cisplatin (EP) and irinotecan/cisplatin (IP) have reported similar efficacy in terms of objective response rates, progression-free survival, and overall survival, though each regimen’s toxicity profile differs [73,74]. Response rates generally range from 40% to 60%, but disease control tends to be transient, with median PFS of approximately 4 to 6 months and median OS around 8 to 13 month [75,76]. In this context, the ongoing French randomized phase II FOLFIRINEC trial (NCT04325425) is testing modified FOLFIRINOX vs. platinum–etoposide as first-line treatment for metastatic GEP-NEC/unknown-primary NEC, results are pending [77]. These findings highlight the urgent need for new treatment strategies and the importance of transitioning to maintenance or second-line therapies following initial cytoreduction.

The SEQTOR study (GET-NE-1206; NCT02246127) is a phase III trial investigating treatment sequencing in advanced pNETs [78]) Patients with progressive, well-differentiated G1–G2 disease were randomized to receive either everolimus first, followed by streptozotocin/5-fluorouracil (STZ/5-FU), or the reverse sequence. Due to slow recruitment, the primary endpoint was adjusted to include 12-month PFS after first-line therapy [79]. Outcomes were broadly similar between sequences (12 m PFS1 69.5% vs. 62.1%; HR 1.2; *p* = 0.43), and median PFS1 did not differ significantly (21.5 vs. 23.6 months). Objective response was higher with first-line STZ/5-FU (30% vs. 11%), supporting chemotherapy when rapid tumor reduction is a priority. However, because 24% of patients were treatment-naïve, SEQTOR does not define the optimal sequence after progression on SSA and should be interpreted accordingly [80] (see Table 2).

In clinical practice, chemotherapy is most appropriate for scenarios requiring rapid tumor reduction, such as pNETs where durable benefit from CAPTEM is predicted.

### 3.4. Peptide Receptor Radionuclide Therapy (PRRT)

Peptide Receptor Radionuclide Therapy (PRRT) utilizing radiolabeled somatostatin analogues has significantly advanced the systemic treatment landscape NETs over the past decade. The beta-emitting radiopharmaceutical Lutetium-177 dotatate (^177^Lu-DOTATATE) received regulatory approval following the pivotal NETTER-1 trial, which demonstrated substantial improvements in PFS for patients with midgut NETs treated with ^177^Lu-DOTATATE in combination with octreotide LAR, compared to high-dose octreotide alone [44]. Currently, PRRT is an established therapeutic option for somatostatin receptor (SSTR)-positive metastatic NETs, particularly following progression on somatostatin analogues (SSAs). The therapy offers meaningful disease control, with a median PFS of approximately 28 months observed in the NETTER-1 long-term follow-up [89], and tumor response rates around 18%. Safety is predominantly hematologic, with laboratory cytopenias frequent but usually transient: lymphopenia 40–45% (grade ≥ 3 ~9%), anemia 60% (grade ≥ 3 1–2%), thrombocytopenia 30–35% (grade ≥ 3 ~2–3%), neutropenia 25–30% (grade ≥ 3 ~3–4%); late therapy-related myeloid neoplasms are uncommon (MDS 2%, AML < 1%), increased in patients who received temozolomide (8–10%) [90]. Non-hematologic AEs are mostly low-grade—nausea 60%, vomiting 45–50%, fatigue 40%—and clinically significant renal toxicity is rare with nephroprotection [91].

Traditionally, PRRT has been reserved for refractory, low- to intermediate-grade NETs. However, emerging evidence supports its application earlier in the treatment course for select cases of aggressive, well-differentiated tumors [43]. The phase III NETTER-2 trial investigated first-line use of ^177^Lu-DOTATATE in patients with newly diagnosed, advanced higher grade 2 and grade3 (Ki-67 index between 10–55%) gastroenteropancreatic NETs. The trial met its primary endpoint, showing that first-line PRRT combined with ongoing octreotide therapy significantly more than doubled median PFS (22.8 months vs. 8.5 months; hazard ratio 0.276, *p* < 0.0001). This 14-month improvement in PFS is clinically meaningful, indicating that even tumors with higher proliferative activity (including well-differentiated grade 3 NETs) can derive considerable benefit from early PRRT. Although the study was open-label, given the logistical challenges of blinding radiotherapy versus injectable therapy; safety profile was consistent with prior PRRT experience, 93–95% of patients experienced at least one adverse event in both treatment arms, with no treatment-related fatalities reported. These findings support, positioning PRRT plus SSA in the upfront management of select high-grade, well-differentiated NETs, a change now reflected in current clinical guidelines [72]. It is important to note that the trial design permitted crossover and subsequent PRRT in patients with progression in the control arm, which may attenuate overall survival differences but nonetheless emphasizes the treatment’s early efficacy.

Another noteworthy phase III trial, COMPETE, directly compared PRRT with molecular targeted therapy. This study evaluated ^177^Lu-edotreotide against everolimus in patients with progressive, grade 1–2 SSTR-positive GEP NETs [43]. The results showed that PRRT significantly prolonged PFS (median 23.9 vs. 14.1 months; HR 0.67, *p* = 0.022). Although OS data remain immature, early analyses suggest a slight, non-significant trend favoring PRRT (approximately 63 vs. 59 months). The safety profile favored PRRT, which was well tolerated with manageable adverse events. These findings (once fully mature) may support regulatory approvals for ^177^Lu-edotreotide and promote earlier use of PRRT in the treatment sequence, potentially after failure of SSAs and before mTOR inhibitors.

In parallel, the phase III COMPOSE trial (NCT04919226) randomizing patients with well-differentiated aggressive grade 2–3, SSTR-positive GEP-NETs to ^177^Lu-edotreotide versus physician’s-choice CAPTEM/FOLFOX or everolimus, remains ongoing, and efficacy results have not yet been reported.

Access to PRRT remains a logistical challenge, requiring specialized facilities, multidisciplinary coordination, and appropriate radiopharmaceutical availability. Variations in healthcare coverage, regional infrastructure, and supply constraints may limit the reach of PRRT, underscoring the importance of equitable access strategies as new agents emerge.

### 3.5. Treatment Sequencing, Access, and Special Populations

With the expanding arsenal of systemic therapies, optimal sequencing in NETs has become a critical consideration. Therapeutic sequencing should be individualized, considering tumor biology parameters, such as grading, differentiation status, proliferation indices, and somatostatin receptor (SSTR) expression, as well as patient comorbidities and prior treatment history, see Figure 1. For instance, in a metastatic well-differentiated midgut NET that exhibits high SSTR expression and is classified as low-grade, an appropriate initial approach might involve the use of SSAs to achieve disease stabilization and symptom control if indicated. Upon disease progression, peptide receptor radionuclide therapy (PRRT) can be considered earlier in the treatment course, especially given emerging evidence from the COMPETE trial supporting its use at this stage [43], with drugs such as everolimus being reserved for later lines of therapy.

In contrast, for pancreatic NET, it may be appropriate to introduce chemotherapy regimens such as capecitabine-temozolomide if tumor reduction is desired. Subsequent lines may include peptide receptor radionuclide therapy or additional targeted agents like cabozantinib, which has demonstrated efficacy even after progression on prior therapies, in particular, in patients previously treated with mTOR inhibitors or PRRT, as shown in the CABINET trial. This trial included many patients with prior everolimus or PRRT exposure, highlighting cabozantinib’s activity in refractory cases [69]. The OCLURANDOM trial comparing ^177^Lu-DOTATATE with sunitinib in pancreatic NETs with prior systemica therapies reported response rates of 63% with PRRT and extended PFS) [83,92].

Given the lack of head-to-head trial comparisons, treatment sequencing decisions are primarily guided by evidence from multiple studies, considering each therapy’s efficacy and toxicity profiles. The emerging data supporting earlier use of PRRT (from NETTER-2 and COMPETE) suggest that, especially for grade 2–3 NETs with high SSTR expression and elevated Ki-67 indices, PRRT should be contemplated at earlier stages rather than as a salvage therapy. This approach offers a non-chemotherapeutic option with favorable PFS prospects [43,81].

On the other hand, cytotoxic chemotherapy still has a key role for certain NETs. For instance, in high-grade tumors or pancreatic NETs requiring significant tumor reduction, chemotherapy regimens such as capecitabine-temozolomide demonstrate a higher overall response rate compared to targeted agents and may be used upfront for tumor debulking [33]. A potential treatment sequence for pancreatic NETs could involve initiating with SSAs for low-burden disease, followed by chemotherapy (such as CapTem) when tumor reduction is necessary, and progressing to peptide receptor radionuclide therapy or other targeted agents, with cabozantinib as a subsequent targeted therapy. For extra-pancreatic NETs, where chemotherapy tends to be less effective, the sequence might involve SSAs, followed by PRRT or mTOR inhibitors like everolimus, and then cabozantinib in later lines.

**Differences across NET subtypes:** Treatment strategies also differ across NET subtypes, reflecting their distinct biological behaviors and responses. Pancreatic NETs often show higher response rates to cytotoxic chemotherapy [33]) and have specific targeted therapies approved (e.g., sunitinib) [68]. They frequently harbor mutations in MEN1, DAXX, or ATRX; however, these alterations have not yet translated into targeted therapies, and current management of pNETs remains based on current available agents.

Midgut (small intestinal) NETs tend to be indolent but eventually metastasize, commonly involving the mesentery and liver [93]; These tumors have high rates of stable disease with SSAs [51] and PRRT, with limited options available beyond these modalities such as mTOR inhibitors (everolimus) [65]. The CABINET trial demonstrated cabozantinib’s efficacy across both pancreatic and non-pancreatic NETs, providing a valuable systemic option for patients with disease progression following SSA and PRRT [69].

Regarding lung neuroendocrine tumors, there is a notable underrepresentation in clinical trials. Everolimus has randomized evidence supporting PFS benefit in nonfunctional lung NETs (RADIANT-4 lung subgroup) and is approved for this indication [65]. By contrast, antiproliferative SSAs and PRRT are not approved for lung NETs, and prospective data are limited. The phase 3 SPINET trial of lanreotide in SSTR-positive bronchopulmonary NETs stopped early for slow accrual and the double-blind PFS difference was not significant [94]. Moreover, SSTR expression in lung NETs is frequently heterogeneous (or absent) on DOTATATE PET [95], restricting PRRT eligibility and potentially attenuating antiproliferative SSA activity in this subtype.

Finally, it is important to recognize the paucity of data regarding NET management in underrepresented populations. Most clinical trials have predominantly involved white participants, and epidemiological evidence indicates that outcomes suggest potential differences; for instance, African American patients with pNETs often present at more advanced stages and may experience slightly worse outcomes [96]. Although there is currently no evidence of differential drug efficacy by race, the limited diversity in clinical trials underscores the need to ensure that therapies are accessible, effective, and generalizable across all patient populations.

## 4. Emerging and Future Directions

### 4.1. Novel Radiopharmaceuticals and Personalized Dosimetry

Building upon the established success of beta-emitting peptide receptor radionuclide therapy (PRRT) with Lutetium-177 DOTATATE, the field is swiftly progressing towards next-generation radionuclide treatments. A particularly promising area involves alpha-particle-emitting radiotherapeutics.

Alpha-emitting isotopes (such as Actinium-225, Lead-212, and Bismuth-213) deliver concentrated energy over very short distances, resulting in dense double-strand DNA damage that may enhance tumor cell eradication while minimizing damage to adjacent normal tissues. Early clinical data for targeted alpha therapy (TAT) in NET are encouraging [44,97].

Current phase III investigations include the ACTION-1 trial, which compares ^225^Ac-DOTATATE to standard treatments such as everolimus, sunitinib, or high-dose somatostatin analogue therapy in patients with G1-G2 GEP NET who have previously received PRRT. The preliminary results from a phase IB component of the trial showed a confirmed objective response rate (ORR) of 29.4% [98]. In this trial, a starting dose of 120 kBq/kg of ^225^Ac-DOTATATE administered every 8 weeks for four cycles was generally well tolerated; however, grade ≥ 3 adverse events occurred in approximately 53% of patients, primarily anemia and lymphopenia, with a subset judged to be treatment-related. Based on safety and tolerability profiles, a fixed dose of 10.2 MBq (275 microcuries) every 8 weeks has been established as the recommended phase 3 dose and is progressing into the randomized phase of the trial [99].

Similarly, an ongoing study (NCT05153772) evaluating Lead-212 labeled antagonists (^212^Pb-DOTAMTATE) has demonstrated significant activity in phase I, with an ORR of 62% in PRRT-naive NET patients and a 56% response rate observed in phase II expansion cohorts [97]. The response has warranted FDA Breakthrough Therapy designation for ^212^Pb-DOTAMTATE as of early 2024. These agents are anticipated to advance into phase II/III trials, such as NCT05636618, planned to assess efficacy in larger patient populations during 2025–2026 [100].

Challenges remain, including the need for comprehensive long-term safety data due to the high-energy nature of alpha particles, which may pose risks of nephrotoxicity or marrow suppression. Production and distribution complexities of alpha-emitting isotopes also present logistical hurdles compared to more established agents like Lutetium-177 [101].

Several novel PRRT studies are evaluating personalized dosimetry, shifting from fixed activity regimens to tailored approaches based on individualized radiation dose assessments. Traditional protocols administer uniform fixed doses, but recent research reveals substantial inter-patient variability in organ and tumor radiation absorption [102]. Trials such as the Phase III DUONEN study are exploring dosimetry-guided strategies, comparing a personalized tandem regimen (combining Lutetium-177 and Yttrium-90 with dose adjustments based on imaging) against conventional fixed-dose protocols. Early data indicate some patients may safely receive higher cumulative activities to maximize tumor response, while others may require treatment de-escalation to minimize organ toxicity [82,103]. Importantly, the dose-limiting compartment may differ between patients (kidney and/or marrow), and current marrow dosimetry remains imperfect; thus, dose escalation or de-escalation should be considered investigational. Observational experience show that some patients tolerate more than 4 doses of ^177^Lu-DOTATATE and may achieve deeper responses or longer PFS; however a survival advantage has not been demonstrated.

This paradigm may evolve into a true theranostic model, where post-imaging dosimetric data guides activity selection for each patient, enabling highly individualized therapy [104,105]. Such approaches also facilitate rational combination therapies, such as pairing PRRT with radiosensitizers or systemic treatments, with close monitoring of organ doses to optimize safety and efficacy [106,107]. Additionally, novel targets beyond somatostatin receptors are under active investigation for tumors lacking SSTR expression. uPAR (urokinase plasminogen activator receptor), often overexpressed in dedifferentiated or SSTR-negative NETs, is a promising candidate, with early-phase trials underway (NCT06980519) to assess radioligand therapies against this target [108].

In summary, the future landscape of neuroendocrine tumor management is expected to incorporate a multipronged arsenal of radionuclide-based therapies, including alpha and beta emitters, receptor agonists and antagonists, delivered in a personalized manner guided by advanced dosimetry and molecular profiling.

### 4.2. Novel Systemic Therapies

Belzutifan

Beyond angiogenesis and mTOR pathways, emerging targeted therapies are being developed. Belzutifan, a first-in-class inhibitor of hypoxia-inducible factor-2α (HIF-2α), represents a novel approach targeting specific tumor biology [109]. While it is not indicated for gastrointestinal NETs, it was recently approved in May 2025 for the treatment of advanced pheochromocytomas and paragangliomas. This approval was based on the results from the phase II LITESPARK-015 trial, which demonstrated an ORR of 26% in patients with unresectable or metastatic pheochromocytoma/paraganglioma. Responses tended to be durable, with a median duration of approximately 20.4 months [110]. Notably, this marks the first oral systemic therapy specifically approved for these rare neuroendocrine tumors.

Belzutifan’s mechanism involves inhibition of HIF-2α, a pathway particularly relevant in tumors driven by von Hippel-Lindau (VHL) pathway mutations. Such mutations are common in certain pheochromocytomas, paragangliomas, and some VHL-associated pNETs. Earlier studies in VHL syndrome have shown that belzutifan can induce tumor regressions across various VHL-associated lesions, with an ORR nearing 90% in indolent VHL-related pNETs [111,112]. However, in sporadic NETs, HIF-2α is not a universal oncogenic driver, limiting the broad applicability of belzutifan in GEP-NETs. Nonetheless, its success in the VHL-associated subset highlights the significance of biomarker-driven therapy selecting patients based on molecular features such as VHL mutations or pseudohypoxia signatures can optimize treatment outcomes.

The development of belzutifan underscores the importance of trial population selection. The pivotal LITESPARK-015 trial was a single-arm study focusing on rare tumors with limited alternative treatments and did not include a control group. Although the ORR of 26% may appear moderate, any tumor response in chemo-refractory pheochromocytomas or paragangliomas is clinically meaningful. The open-label design means interpretations rely on comparison with historical data; however, the prolonged responses and observed improvements in blood pressure in some patients provide encouraging evidence of efficacy. Currently, belzutifan is considered a new standard of care for advanced pheochromocytoma and paraganglioma, replacing systemic chemotherapy in this setting. Access may be restricted outside specialized centers due to rarity and cost considerations. For patients who progress after belzutifan, options such as PRRT in SSTR-positive cases or cytotoxic regimens like cyclophosphamide-vincristine-dacarbazine (CVD) remain available.

Immunotherapy

Despite broad success in various malignancies; immune checkpoint inhibitors (ICIs) have shown limited activity in most well-differentiated NETs [113,114]; which often have low TMB, scarce PD-L1 expression, and an immune-desert microenvironment. Clinical trials evaluating PD-1/PD-L1 inhibitors in unselected NET populations have confirmed these limited effects. For example, the KEYNOTE-158 study assessing pembrolizumab monotherapy in previously treated NETs reported an ORR of approximately 3.7% (4 responders out of 107 patients). The median PFS was around 4.1 months, comparable to historical placebo data, with no improvement in overall survival [115]. Accordingly, ICIs are not standard care for most well-differentiated NETs and are generally reserved for rare, biomarker-defined scenarios (e.g., MSI-H/dMMR or TMB-high disease), for which pembrolizumab holds tissue-agnostic approvals [116].

Therapy-induced hypermutation has been described after alkylating agents (and sometimes in the setting of prior PRRT exposure): in a prospective, multinational cohort about one-third of progressing NENs met TMB-high thresholds and more often harbored MMR defects; many cases also showed grade progression from G2 to G3 [117]. Importantly, TMB-high alone may not ensure ICI benefit; however, emerging retrospective data suggest that PanNETs with alkylating-induced TMB-high and/or acquired MMR alterations can have higher response rates and longer PFS with ICI than biomarker-negative counterparts (e.g., ORR ~30% vs. 0% in TMB-high vs. low/unknown; ORR ~42% vs. 7% in MMR-altered vs. no/unknown), findings that remain hypothesis-generating pending prospective validation [118]. Overall, ICI use in well-differentiated NETs should be selective and biomarker-guided, ideally within trials.

A more promising area for immunotherapy appears in high-grade neuroendocrine neoplasms (NENs), particularly poorly differentiated neuroendocrine carcinomas. These aggressive tumors, often share genomic and microenvironmental features with small-cell lung cancer (such as higher mutational burdens) which may render them more susceptible to immunotherapeutic strategies. The combination of CTLA-4 and PD-1 blockade has shown activity in small studies. The phase II DART trial, a National Cancer Institute-sponsored basket study of rare tumors, identified a cohort of high-grade NETs/NECs treated with nivolumab plus ipilimumab. The non-pancreatic high-grade NEN subgroup, reported a post hoc ORR of 44% vs. 0% observed in intermediate/low-grade NETs. However, in the subsequent dedicated high-grade cohort of DART, the prospectively evaluated ORR was 26%, with modest median PFS/OS [119]. Taken together these findings suggest that dual checkpoint inhibition could be considered for select patients with high-grade NEN/NEC, acknowledging the limitations of small sample sizes and non-prespecified subgroup analyses [120].

Ongoing clinical trials are further assessing nivolumab with or without ipilimumab in advanced extrapulmonary neuroendocrine carcinomas (e.g., NCT05262556). In clinical practice, some oncologists consider nivolumab and ipilimumab as second-line options for metastatic NEC following platinum-based chemotherapy, particularly in cases with high PD-L1 expression or limited alternative treatments [121,122]. Nonetheless, these data derive from relatively small cohorts.

DLL3 is frequently expressed in neuroendocrine carcinomas (NEC), notably small-cell and large-cell variants, while being uncommon in well-differentiated GEP-NETs, supporting DLL3 as a therapeutic target primarily in NEC rather than NET [85]. Obrixtamig (BI 764532), a DLL3/CD3 bispecific T-cell engager, has shown promising activity and a manageable safety profile in an ongoing phase 1 trial (NCT04429087). The DAREON clinical program is now testing this agent in later-phase settings: DAREON-5 (NCT05882058) is an open-label, multicenter phase 2 dose-selection study in relapsed/refractory SCLC, LCNEC, and extrapulmonary NEC, and DAREON-7 is a phase 1 trial combining BI 764532 + platinum/etoposide in first-line DLL3-positive NEC. Early reports (including 2025 updates from the phase 1 cohort) describe objective responses in epNEC that appear higher than typical post-platinum benchmarks, but randomized data are pending; thus, DLL3-directed therapy remains investigational pending mature efficacy and survival readouts [85,86,87,88].

For well-differentiated NETs, current research efforts focus on strategies to overcome resistance to immunotherapy. Early-phase trials are investigating combinations of anti–PD-1 agents with VEGF inhibitors or chemotherapy [121,123]. Considerable scientific interest is also directed towards modifying the tumor microenvironment, converting immunologically “cold” tumors into “hot” ones, more amenable to immune attack [124]. Novel combinations targeting other immune checkpoints, such as anti-LAG-3 or anti-TIM-3 antibodies, are under exploration in phase II studies, particularly for NETs with higher proliferative indices, such as pulmonary atypical carcinoids with high tumor-infiltrating lymphocytes or MMR-deficient NETs, that might benefit from immunotherapy [125,126].

Oncolytic viruses and cancer vaccines are under investigation as strategies to “prime” an immune response in NETs. For example, a phase I trial is evaluating the oncolytic Seneca Valley virus (SVV-001) in combination with nivolumab and ipilimumab for high-grade NENs, with the aim of inflaming the tumor microenvironment and enhancing responsiveness to immunotherapy [127]. While innovative, these approaches remain highly preliminary as of 2025.

CAR T-cell therapy

Given modest ICI efficacy in many NETs, highly specific strategies are advancing. Chimeric Antigen Receptor (CAR) T-cell therapy, which has demonstrated significant success in hematologic malignancies through targeting lineage-specific antigens, is being explored for solid tumors, including NETs. This approach needs the identification of tumor-specific, safe surface targets with high expression levels on NET cells [128]. One promising target is Cadherin-17 (CDH17), an adhesion molecule expressed predominantly in gastrointestinal NETs and certain other GI cancers, but not widely present on normal adult tissues [129]. The U.S. Food and Drug Administration (FDA) approved the investigational new drug (IND) application for the first clinical trial of CDH17-targeted CAR T cells (product code CHM 2101) in patients with advanced neuroendocrine, colorectal, or gastric cancers [130].

Another investigational target is IL13 receptor α2 (IL13Rα2), which is expressed on a subset of metastatic NETs. An ongoing phase I trial is evaluating IL13Rα2-specific CAR T cells in solid tumors, including NETs, building on prior applications in melanoma and glioma. These early-stage studies mark the inaugural exploration of CAR T-cell therapy in NET patients, highlighting a notable development in 2025 [131].

Optimizing CAR T-cell strategies will require time and rigorous clinical validation. Complementary immunotherapy approaches are also in development, including bispecific T-cell engagers (BiTEs) and antibody-drug conjugates targeting NET-associated antigens such as somatostatin receptor 2 (SSTR2) and asialoglycoprotein receptor 1 (ASGR1) [132,133,134].

### 4.3. DNA Damage Response Inhibition as a Radiosensitizer

Inhibiting DNA repair mechanisms is another promising strategy to enhance radiotherapy efficacy. The LuPARP phase I trial evaluated oral olaparib (50–300 mg BID) alongside standard ^177^Lu-DOTATATE therapy, indicating feasibility and manageable toxicity, with some patients experiencing partial responses, including those previously progressed on somatostatin analog therapy [31]. Additional agents targeting DNA repair—such as triapine, are under investigation in randomized trials, aiming to improve therapeutic responses by increasing tumor radiosensitivity (NCT05724108) [135]. Likewise, chemotherapeutic combinations like CAPTEM continue to demonstrate significant activity, with ongoing studies (phase II randomized study CAPRRI-NET) seeking to confirm whether adding these agents to PRRT can improve progression-free survival [136].

### 4.4. Adaptive and Biomarker-Enriched Clinical Trials

Given the heterogeneity and rarity of NETs, innovative trial designs such as adaptive methodologies enable more efficient evaluation of new treatments. The CABINET trial for cabozantinib is an example, leveraging interim analyses to identify significant efficacy early, facilitating regulatory review and patient access [69]. Basket trials are increasingly used to include NET patients harboring actionable molecular alterations, such as BRAF V600E mutations or NTRK fusions, within broader genotype-driven studies.

Molecular profiling and biomarker development are shaping personalized treatment strategies. Multi-omic profiling of pancreatic NETs segregates tumors by lineage factors (ARX- vs. PDX1-driven) and metastatic potential, offering a rationale for escalating therapy intensity in the “metastatic” transcriptomic cluster [137]. Complementing this, the first NET organoid biobank couples genomics, epigenomics and pharmacologic screens, generating patient-specific drug-response signatures that can inform trial stratification [138]. Furthermore, methylation status of the MGMT promoter helps predict responsiveness to temozolomide-based regimens and is incorporated into trial enrichment criteria [33].

Prospective studies like SEQTOR and COMPETE are exploring optimal sequencing of therapies (e.g., PRRT versus targeted agents) [139]. Trials such as STARTER-NET in Japan (JCOG1901) evaluated everolimus + lanreotide vs. everolimus in untreated G1–G2 GEP-NETs with poor-risk features: the combination prolonged PFS (interim 29.7 vs. 11.5 months; updated 29.7 vs. 13.6 months), but OS is immature and many experts view these findings as confirmatory of lanreotide activity rather than practice-changing at present [84]. By mid-2025, pivotal readouts such as NETTER-2 and COMPETE were informing earlier use of PRRT in selected patients and refining positioning vs. targeted agents; however, genomic-driven sequencing remains investigational, and current decisions are best anchored in grade, SSTR expression, disease tempo/burden, prior therapy, toxicity profiles, and patient preferences (see Table 2).

## 5. Conclusions

The treatment landscape for neuroendocrine tumors in 2025 is characterized by unprecedented advancements and ongoing innovation. State-of-the-art diagnostic modalities, including multi-analyte liquid biopsies and next-generation somatostatin receptor PET imaging, now enable earlier detection of occult disease, refined biological risk stratification, and precise identification of candidates PRRT.

Therapeutic strategies have evolved into a nuanced, tiered approach. Somatostatin analogues remain the cornerstone of first-line therapy for well-differentiated tumors, with targeted agents such as everolimus and sunitinib continuing to play vital roles in pancreatic neuroendocrine neoplasms. The regulatory approval of ^177^Lu-DOTATATE has established PRRT as the standard radionuclide treatment for SSTR-positive gastrointestinal and pancreatic tumors, with ongoing trials exploring its efficacy in earlier treatment lines and higher-grade disease. Notably, cabozantinib has demonstrated phase III efficacy across both pancreatic and extrapancreatic cohorts, addressing a longstanding unmet need by providing a broadly effective multikinase inhibitor in patients previously treated with targeted therapies or PRRT. Additionally, belzutifan introduces a novel mechanism, targeting hypoxia pathways, showcasing biomarker-driven drug development in rare neuroendocrine tumor subtypes such as pheochromocytoma and paraganglioma.

Emerging innovations are poised to further enhance patient outcomes. The development of somatostatin receptor antagonists and alpha-particle emitting PRRT platforms are anticipated to improve response depth and durability, especially when combined with personalized dosimetry. Recent phase 3 readouts such as NETTER-2 and COMPETE are helping to refine the positioning of PRRT relative to targeted agents, and ongoing randomized studies of dosimetry-guided PRRT may inform future practice once mature results are available.

Despite these advances, challenges remain. Disparities in access to advanced imaging, PRRT, and high-cost oral agents persist globally. Representation of diverse populations—including lung, hindgut, pediatric, and racial minorities—in clinical trials remains limited. Moreover, predictive biomarkers to inform optimal treatment sequencing are still being validated. Overcoming these hurdles will require inclusive, multiomic trial designs, standardized dosimetry protocols, and coordinated international efforts to expand treatment infrastructure.

The integration of advanced diagnostics with mechanism-specific therapies continues to transform the management paradigm for both pancreatic and extra-pancreatic neuroendocrine tumors. Sustained multidisciplinary collaboration, equitable resource distribution, and rigorously designed biomarker-driven research are essential to translating these scientific advancements into meaningful improvements in survival and quality of life for all individuals affected by neuroendocrine neoplasms.

## Figures and Tables

**Table 1:** Approved systemic therapies in NETs (as of 2025).

Therapy (Mechanism)	Indication (NET Subtype/Line)	Supporting Trial (s) (N)	Efficacy Outcomes	Critical Commentary
Octreotide LAR/Lanreotide (SSTR agonists)	Well-differentiated G1–G2 GEP-NETs; first-line for tumor control and symptom control	PROMID (midgut) N = 85; CLARINET (non-functioning enteropancreatic, Ki-67 ≤10%) N = 204.	PROMID: TTP 14.3 vs. 6.0 mo (HR 0.34). CLARINET: 24-mo PFS 65% vs. 33%; median PFS NR vs. 18.0 mo.	Antiproliferative effect strongest in low-volume, low-Ki-67 disease; limited tumor shrinkage; OS effect confounded by crossover. Not studied for G3 WD disease.
Everolimus (mTOR inhibitor)	Progressive G1–G2 pNET; non-functioning GI/lung NET after SSA or upon progression	RADIANT-3 (pNET) N = 410; RADIANT-4 (GI/lung NET) N = 302.	RADIANT-3: PFS 11.0 vs. 4.6 mo (central). RADIANT-4: PFS 11.0 vs. 3.9 mo; HR ~0.48–0.59 across analyses; ORR low.	Reliable PFS benefit across prior-therapy strata; ORR modest; OS neutral due to crossover. Mucocutaneous and metabolic AEs require monitoring.
Sunitinib (VEGFR/PDGFR inhibitor)	Progressive, well-differentiated pNET	Phase III SUN 1111 (pNET) (2011) N = 171	PFS: 11.4 mo vs. 5.5 mo on placebo (HR 0.42, *p* < 0.001).	pNET-specific evidence; trial halted early, but effect size and consistency support use. Hypertension, fatigue, diarrhea common.
Cabozantinib (Multi-kinase inhibitor: VEGFR2, MET, AXL)	pNET and extra-pancreatic NET after prior therapy	CABINET (Phase III, 2025) N = 298	pNET: PFS 13.8 vs. 4.4 mo (HR 0.23); ORR 19%. epNET: PFS 8.4 vs. 3.9 mo (HR 0.38); ORR 5%.	Expands kinase options in both pNET and epNET; clinically meaningful PFS with low ORR typical of antiangiogenic TKIs; grade ≥ 3 AEs ~60% require dose adjustment. OS not yet clearly improved (crossover).
Capecitabine + Temozolomide (CAPTEM) (Cytotoxic chemotherapy)	Advanced pNET (often used second-line or later)	ECOG-ACRIN E2211 (Phase II randomized) N = 144	PFS 22.7 vs. 14.4 mo (HR 0.58); OS 58.7 vs. 53.8 mo (NS). MGMT deficiency associated with higher response.	Only randomized evidence is phase II; nevertheless, robust PFS and response support use. Consider MGMT testing to enrich benefit; myelosuppression and nausea manageable with standard prophylaxis.
^177^Lu-DOTATATE (PRRT) (beta-emitting radioligand)	SSTR-positive WD GEP-NETs: (A) midgut after progression; (B) first-line for higher-grade WD (G2–G3) per NETTER-2	NETTER-1 (midgut) N = 229; NETTER-2 (first-line G2–G3 WD GEP-NETs) N = 226.	NETTER-1: median PFS NR vs. 8.4 mo; HR 0.21; ORR 18% vs. 3%. NETTER-2: PFS 22.8 vs. 8.5 mo; HR 0.28.	Strongest randomized PFS evidence in SSTR-positive disease; hematologic/renal toxicity usually low-grade with amino-acid protection. OS interpretation limited by crossover. First-line adoption for G2–G3 will depend on guideline updates and access.

Abbreviations: AE, adverse event; epNET, extra-pancreatic NET; GEP-NET, gastroenteropancreatic NET; HR, hazard ratio; LAR, long-acting release; NR, not reached; ORR, objective response rate; OS, overall survival; pNET, pancreatic NET; PFS, progression-free survival; PRRT, peptide receptor radionuclide therapy; SSA, somatostatin analogue; SSTR, somatostatin receptor; TTP, time to tumor progression; WD, well differentiated.

**Table 2:** Pivotal and Emerging Clinical Trials Shaping the NET Therapeutic Landscape.

Trial (Phase)—Intervention	Population	Design/Primary Endpoint	Results or Status (Mid 2025)
COMPETE (Phase III)—^177^Lu-edotreotide vs. Everolimus [81]	Metastatic SSTR-positive G1–G2 GEP-NETs; no prior PRRT (pNET and non-pNET)	Open-label RCT; primary: PFS (central review)	Positive. PFS 23.9 vs. 14.1 mo, HR 0.67, *p* = 0.022; ORR 16% vs. 5%; fewer grade 3–4 AEs with PRRT.
DUONEN (Phase III)—standard ^177^Lu-DOTATATE vs. dosimetry-guided ^177^Lu/^90^Y tandem regimens [82]	Advanced WD NETs (G1–G2), SSTR-positive, progressed on SSA	Multi-center, 4-arm trial; standard 7.4 GBq ^177^Lu × 4 vs. mixed ^177^Lu/^90^Y (increasing ^90^Y by dosimetry); primary: PFS	Ongoing. Interim safety acceptable; tests whether personalization (incl. ^90^Y for larger lesions) improves tumor control/PFS.
OCLURANDUM (Phase III)— ^177^Lu-DOTATATE vs. Sunitinib [83]	Advanced pancreatic NET (G1–G2), progressed on SSA	Open-label RCT; primary: 12-mo PFS (non-inferiority → superiority); crossover allowed at progression	Efficacy signal for PRRT. ORR 63% vs. 30%; median PFS 20.7 vs. 11.0 mo. OS numerically longer with sunitinib (64.4 vs. 55.8 mo) likely from crossover; PRRT better tolerated.
STARTER-NET/JCOG1901 (Phase III)—Everolimus + Lanreotide vs. Everolimus [84]	Unresectable GEP-NET (G1–G2), first-line systemic therapy	Randomized 2-arm; primary: PFS; stratified by primary site	Interim positive. PFS 29.7 vs. 11.5 mo, HR 0.38, *p* < 0.001; ORR higher with combo (~18% vs. 5%); OS immature; toxicity manageable.
SEQTOR (GETNE-1206)—Phase III Everolimus → STZ/5-FU vs. STZ/5-FU → Everolimus [79]	Advanced, progressive pNET (G1–G2); systemic-therapy-naïve	Open-label 2-arm sequence study; primary (amended): 12-mo PFS after first-line (PFS1); key secondary: PFS1 + PFS2, ORR, OS, safety	Similar disease control either way (PFS1 21.5 vs. 23.8 mo). Higher ORR when STZ/5-FU first (30% vs. 11%).
STELLAR-311 (Phase II/III)—Zanzalintinib (XL092) vs. Everolimus [NCT06943755]	Unresectable/metastatic WD (G1–G3) pNET/epNET	Randomized phase II/III; primary: PFS (central review); key secondary: ORR, OS, safety.	Ongoing.
DAREON-5 (Phase II)—Obrixtamig (BI 764532) [85,86,87,88]	DLL3-positive extrapulmonary high-grade NEC (incl. LCNEC/GEP-NEC), relapsed/refractory	Open-label, multicenter dose-selection; primary: ORR (RECIST v1.1); secondary: DOR, PFS, safety	Ongoing. Early-phase data show confirmed responses; no randomized data yet.

Abbreviations: AE = adverse event; DOR = duration of response; epNET = extra-pancreatic neuroendocrine tumor; GEP-NET = gastroenteropancreatic neuroendocrine tumor; G1/G2/G3 = WHO grade 1/2/3; LCNEC = large-cell neuroendocrine carcinoma; NEC = neuroendocrine carcinoma; NET = neuroendocrine tumor; ORR = objective response rate; OS = overall survival; pNET = pancreatic neuroendocrine tumor; PFS = progression-free survival; PFS1 = first-line PFS; PRRT = peptide receptor radionuclide therapy; RCT = randomized controlled trial; RECIST v1.1 = Response Evaluation Criteria in Solid Tumors version 1.1; SSA = somatostatin analogue; SSTR = somatostatin receptor; STZ/5-FU = streptozotocin + 5-fluorouracil; WD = well-differentiated; 177Lu = Lutetium-177; 90Y = Yttrium-90; DLL3 = delta-like ligand 3.

## Data Availability

No new data were created or analyzed in this study. Data sharing is not applicable to this article.
